# Molecular mechanisms of pyroptosis and its role in anti-tumor immunity

**DOI:** 10.7150/ijbs.86855

**Published:** 2023-08-06

**Authors:** Hongyong Huang, Yanmin Weng, Wen Tian, Xian Lin, Jian Chen, Lianxiang Luo

**Affiliations:** 1 The First Clinical College, Guangdong Medical University, Zhanjiang, 524023, Guangdong, China.; 2 Department of Rheumatism and Immunology, Peking University Shenzhen Hospital, Shenzhen, Guangdong, 518036, China; Shenzhen Key Laboratory of Inflammatory and Immunology Diseases, Shenzhen, Guangdong, 518036, China.; 3 The Marine Biomedical Research Institute, Guangdong Medical University, Zhanjiang, 524023, Guangdong, China.; 4 The Marine Biomedical Research Institute of Guangdong Zhanjiang, Zhanjiang, 524023, Guangdong, China.

**Keywords:** pyroptosis, tumor microenvironment, Gasdermin, inflammasome, antitumor immunity

## Abstract

Pyroptosis is a form of cell death that is characterized by the destruction of the cell, and it has implications in both the immune system and cancer immunotherapy. The gasdermin family is responsible for the activation of pyroptosis, which involves the formation of pores in the cellular membrane that permit the discharge of inflammatory factors. The inflammasome response is a powerful mechanism that helps to eliminate bacteria and cancer cells when cellular damage occurs. As tumor cells become more resilient to apoptosis, other treatments for cancer are becoming more popular. It is essential to gain a thorough understanding of pyroptosis in order to use it in cancer treatment, considering the intricate association between pyroptosis and the immune system's defensive reaction against tumors. This review offers an overview of the mechanisms of pyroptosis, the relationship between the gasdermin family and pyroptosis, and the interplay between pyroptosis and anti-tumor immunity. In addition, the potential implications of pyroptosis in cancer immunotherapy are discussed. Additionally, we explore future research possibilities and introduce a novel approach to tumor treatment.

## Introduction

Pyroptosis, also known as a type of programmed cell death (PCD), is vital to immune responses in the human immune system. As part of innate immunity, PCD not only contributes to guarding against pathogens, but also to eliminating tumor cells, which has attracted the attention of a growing number of scientists. Based on changes in cell morphology, PCD is usually divided into lytic and nonlytic cell death^1^. Apoptosis is a relatively "quiet" immune clearance called non-lytic PCD, while pyroptosis is a relatively "robust" clearance called lytic PCD. Inflammasomes activation and the release of various pro-inflammatory factors are two extremely crucial processes in pyroptosis to guard against infections. Generally, gasdermins (GSDMs), the executor of pyroptosis, have been believed to exist as a novel therapeutic target for the development of cancer medications^2^.

Pyroptosis was first used in 2001 to describe a unique way of PCD^3^. Pyroptosis is characterized by cell swelling and volume enlargement. When swelling reaches a certain level, the cells rupture and break down^4^. Researchers have demonstrated that there are a large number of bubble-like protrusions related to GSDMD on the cell membrane^4^. Gasdermine D (GSDMD) is cleaved into a C-terminal domain (GSDMD-C) and an N-terminal domain (GSDMD-N) translocated to the cell membrane to form oligomers, also known as "pyroptotic bodies". The cell membranes form non-selective ion-permeable pores through "pyroptotic bodies", allowing small molecules to pass, resulting in an unbalanced osmotic pressure inside and outside the cell, giving rise to water inflow, usually accompanied by ions outflow^5^-^7^.

Morphologically, pyroptosis shares feature with apoptosis and necrosis. Similar to apoptosis, pyroptosis is characterized by nuclear shrinkage resulting from DNA damage^8^, ^9^. However, distinct from apoptotic cells, pyroptotic cells maintain mitochondrial integrity and cytochrome c is not released^5^, ^6^. Similar to necrosis, cell membrane integrity is disrupted. Cytoplasmic inflammatory contents containing mature interleukin-1β (IL-1β) and interleukin-18 (IL-18) are rapidly released and induce an inflammatory response^8^. Distinct from necrosis, pyroptosis is usually mediated by caspase-1 associated with the release of IL-1β and IL-18^5^. Further research related to pyroptosis has shown that other caspases also mediate pyroptosis, and pyroptosis is usually dependent of caspases^10^.

Recent investigations have demonstrated that pyroptosis is strongly linked to anti-tumor immunity and inflammatory reactions. Pyroptosis has the capacity to impede cancer growth, invasion, and metastasis, yet it may also have a tumor-promoting effect in a number of cancers. Consequently, pyroptosis may be employed in novel approaches for cancer therapy. Pyroptosis is a molecular mechanism that is involved in anti-tumor immunity. This process involves the activation of caspases, resulting in the release of pro-inflammatory cytokines and other molecules. We likewise examine the use of pyroptosis in cancer therapy, as well as its potential future directions.

## The molecular mechanisms of Pyroptosis

**Figure 1:**
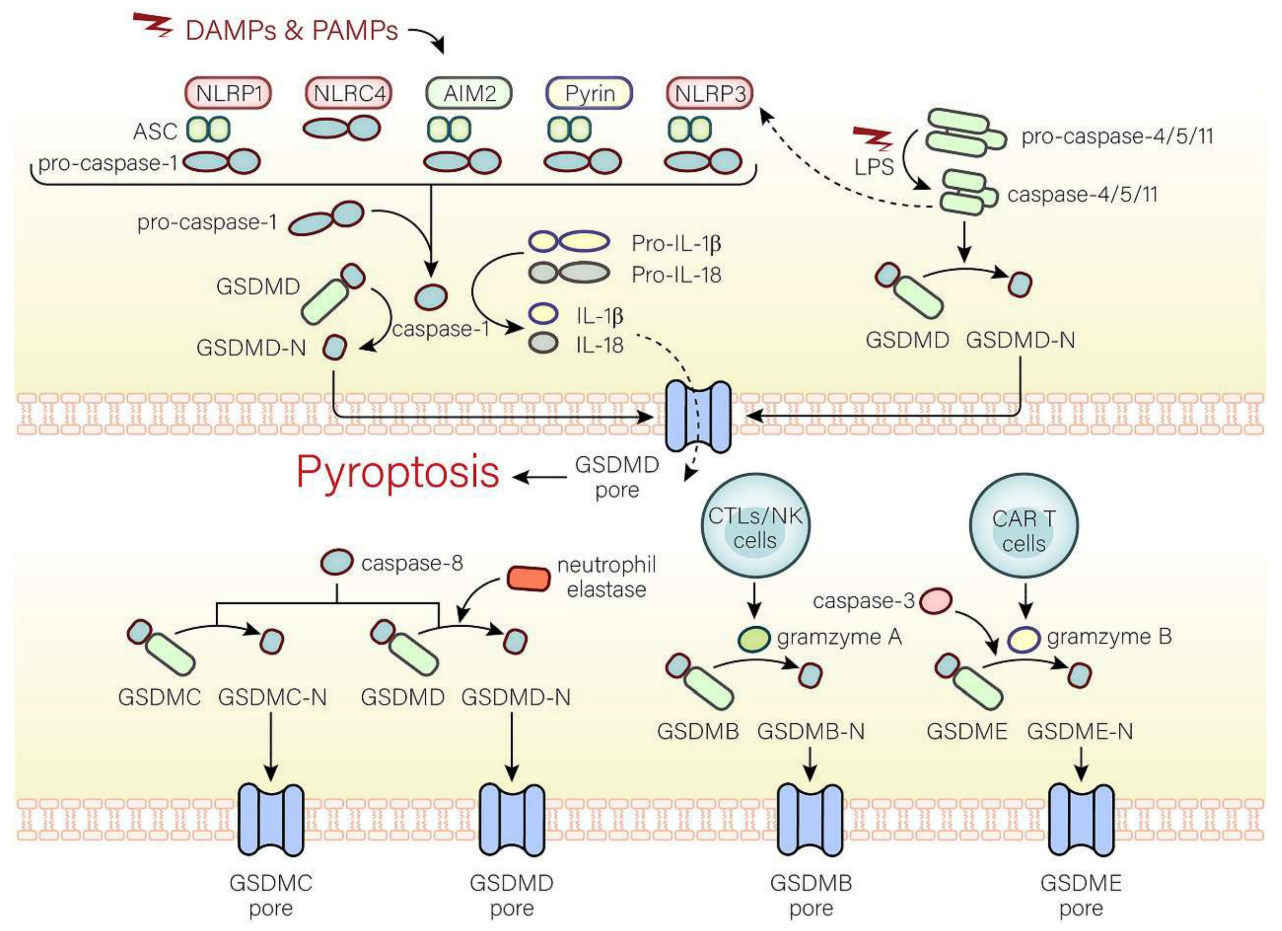
** The molecular mechanisms of pyroptosis.** In the canonical pathway, the canonical inflammasome pathway (NLRP1, NLRP3, NLRC4, AIM2, Pyrin) is activated and assembled first. Subsequently, the classical inflammasome triggers GSDMD-dependent pyroptosis through caspase-1. In the non-canonical pathway, the non-canonical inflammasome activated by LPS begins to assemble and also trigger GSDMD-dependent pyroptosis. Among alternative signaling pathways, CTLs, NK cells, and CAR T cells can deliver granzymes to target cells through the pores formed by perforin on target cells, thereby cleaving GSDMs, and triggering pyroptosis. Caspase-3 and caspase-8 triggers pyroptosis through pores on the cell membrane which are caused by GSDME-N and GSDMC-N respectively. Neutrophil elastase can mediate GSDMD-dependent pyroptosis.

Pyroptosis has multiple pathways through which it can be initiated, such as the standard inflammasome pathway, the non-canonical inflammasome pathway, and the alternative signaling pathways (Fig. 1).

### The canonical inflammasome pathway

The canonical inflammasome pathway is mediated by pattern-recognition receptors (PRRs), including NOD-like receptor (NLR) family pyrin domain-containing (NLRP1), NLR protein 3 (NLRP3), NLR protein C4 (NLRC4), absent in melanoma 2 (AMI2), and pyrin, which can identify pathogen-associated molecular patterns (PAMPs) or damage-associated molecular patterns (DAMPs). PRRs are stimulated and activated, recruiting caspase-1 and combining with caspase-1 and an adaptor protein (ASC), both of which are assembled into inflammasomes^11^, ^12^. Caspase-1, the key to pyroptosis in the canonical inflammasome pathway, is activated by inflammasomes and cleaves not only pro-IL-18 and pro-IL-1β to form active IL-18 and IL-1β, but also GSDMD into GSDMD-C and GSDMD-N^11^. GSDMD-N binds to phospholipid proteins on cell membranes, forming pores and triggering pyroptosis and inflammatory responses^4^, ^11^. However, there are huge differences in pyroptosis induced by different inflammasomes. In the following, we will discuss the activation of inflammasomes.

NLRP1 can form inflammasomes that trigger pyroptosis and inflammatory reactions^13^. The human genomes only contain NLRP1 gene, while three NLRP1 orthologs (NLRP1a, NLRP1b, NLRP1c) are present in mice^14^. After pathogen recognition, NLRP1, NLRP1a, or NLRP1b subsequently assembles with ASC, and pro-caspase-1 via caspase recruitment domain (CARD) to form the NLRP1 inflammasome, whereas ASC isn't essential to the NLRP1 inflammasome in mice^15^-^17^. In addition, the inhibition of the dipeptidyl peptidases 8 and 9 (DPP8 and DPP9), cytosolic serine dipeptidyl peptidases, serves to activate and assemble the NLRP1b inflammasome, thus triggers caspase-1-dependent pyroptosis^17^.

The NLRP3 inflammasome is characterized by NLRP3, ASC, and pro-caspase-1 and activated by two pathways^18^. In the typical activation of the NLRP3 inflammasome, two key signals, namely, the initiation signal (signal 1) and the activation signal (signal 2), are necessary. The function of signal 1 is to increase the NLRP3 and pro-IL-1β expression level, whereas the function of the activation signal is to promote the formation of the NLRP3 inflammasome. With permission from signal 1, signal 2 can work. In signal 1, after Toll-like receptor 4 (TLR4) receptors, the myeloid differentiation primary response 88 (MyD88) receptors, or time-restricted feeding (TRF) receptors accept the stimulation of microorganisms or the effect of pro-inflammatory factors, NLRP3 and pro-IL-1β transcription will increase through the IL-1 receptor-associated kinase (IRAK) or nuclear factor-kappa B (NF-κB) pathway^19^, ^20^. Different NLRP3 activators (such as mitochondrial reactive oxygen species (ROS) production, cholesterol crystals, calcium mobilization, etc.) can trigger signal 2^19^-^22^. In the pathway of the non-canonical NLRP3 inflammasome activation, caspase-4/5 in humans and caspase-11 in mice mediate the activation of NLRP3 inflammasome^23^-^28^.

The NLRC4 inflammasome can be activated by Salmonella, the flagellin of the pathogen Legionella pneumophila, and the rod-shaped portion of the type III secretion system (TTSS). Notably, flagellin and TTSS rod-shaped proteins are not directly recognized by NLRC4 but by NAIP and apoptosis inhibitory proteins in the NLR family^29^-^31^.

AIM2 is believed to exist as a DNA sensor that recognizes cytoplasmic DNA, especially double-stranded DNA^32^, ^33^. Notably, only dsDNA of at least 80 bp in length can connect to the HIN-200 domain^34^, ^35^. Negatively-charged bacterial dsDNA can replace the PYD domain of AIM2 and bind to the positively-charged HIN-200 domain through electrostatic effects, activating the PYD domain. AIM2 is subsequently assembled with ASC and caspase-1 to form the AIM2 inflammasome^33^, ^34^, ^36^, ^37^.

Pyrin can indirectly detect inactive proteins under the action of many bacteria^38^, ^39^. In humans, Protein Kinase N1 (PKN1) and Protein Kinase N2 (PKN2), members of the protein kinase C (PKC) superfamily of kinases, are activated by Ras homolog gene family member A (RhoA), connect to human pyrin and phosphorylate S208 and S242, which are subsequently assembled with 14-3-3ε or 14-3-3τ to inhibit pyrin activation^39^. In mice, the combination between phosphorylated Ser-205 and Ser241 and the 14-3-3 protein inhibits pyrin activation^40^. However, with the inactivation of the Rho-modified protein, the interaction between the 14-3-3 protein and pyrin is reduced, and the inhibitory effect is reduced, promoting the activation and assembly of the pyrin inflammasome that is characterized by pyrin, ASC, and caspase-1^41^, ^42^.

### The non-canonical inflammasome pathway

The caspase-4/5/11-LPS complex is believed to exist as the non-canonical inflammasome that mediates the non-canonical pathway. In the cytoplasm, pathogenicity-associated lipopolysaccharides (LPS) of some gram-negative bacteria can bind to caspase-4/5/11 without inflammasomes by CARD fragments^28^. In addition, CARD fragments of caspase-4/5 are in humans and CARD fragments of caspase-11 are in mice^28^. Moreover, caspase-4 activation is dependent on guanylate-binding proteins that recruit and activate caspase-4 at the bacterial membranes, while caspase-11 activation is mediated by high mobility group box 1 (HMGB1) 43, 44. The activated caspase-4/5/11 cleave GSDMD at ASP276 and subsequently release the GSDMD-C fragment and GSDMD-N pore-forming fragment that disrupts the membrane integrity and triggers pyroptosis. In addition, the non-canonical inflammasome can also activate the NLRP3 inflammasome, suggesting that the non-canonical pathway is associated with the canonical pathway^23^, ^25^, ^28^. However, the relationship between the non-canonical pathway and the canonical pathway is still unknown and worth investigating.

### Alternative signaling pathways

Pyroptosis can also be activated by other pathways. Caspase-3 induces pyroptosis through the pores on the cell membrane caused by gasdermine E (GSDME)^45^. During Yersinia infection, the receptor interacting protein 1 (RIPK1)-caspase-8 complex cleaves GSDMD and induces pyroptosis dependent on Folliculin-Folliculin-interacting protein2-Rag-Ragulator complex^46^. Moreover, active caspase-8 can cleave oxidized death receptor DR6-dependent gasdermine C (GSDMC) to form GSDMC-N segments and cause pyroptosis^47^. Besides caspases, pyroptosis can be mediated by other substances. GSDMD cleavage in neutrophils is dependent on neutrophil elastase and involved in pyroptosis^48^. In cells undergoing apoptosis, cytotoxic T lymphocytes (CTLs) and natural killer (NK) cells can produce granzyme A (GZMA) that transports into cells. If gasdermine B (GSDMB) is present in the cytoplasm, GSDMB will be cleaved by GZMA into GSDMB-N-terminal to trigger pyroptosis^49^. In addition, granzyme B (GZMB) released from chimeric antigen receptor (CAR T) cells mediates the cleavage of GSDME to cause pyroptosis^50^.

#### Gasdermin family and pyroptosis

GSDMs, a family of intracellular proteins, can mediate pyroptosis. Six GSDMs proteins (GSDMA, GSDMB, GSDMC, GSDMD, GSDME, DFNB59) are present in the human system, while ten GSDMs proteins (GSDMA1, GSDMA2, GSDMA3, GSDMC1, GSDMC2, GSDMC3, GSDMC4, GSDMD, GSDME, DFNB59) are edited in mice^51^, ^52^. The Gasdermin family (except DFNB59) consists of the C-terminal inhibitory domain associated with the intermediate transition region and the N-terminal domain that binds to lipids in the cell membrane to generate pores, interrupt the integrity of cell membrane integrity and liberate cellular contents, resulting in the extracellular release and pyroptosis^52^, ^53^. Of note, GSDMD is regarded as the strongest substrate of inflammatory caspases and the only caspase-1 substrate for inducing pyroptosis^54^-^57^. In mouse D276 and human D275, the linker between GSDMD-C and GSDMD-N has a caspase-1-targeted cleavage site that is activated by the downstream inflammasome complexes. After cleavage, this weakens the inhibitory of GSDMD-C on GSDMD-N that freely oligomerizes at the cell membrane, forming pyroptosis-inducing pores and triggering pyroptosis^58^.

#### Immune cells and pyroptosis

The immune system is characterized by innate and adaptive immunity and believed to exist as a defense against pathogens and a useful tool to eliminate cancer cells. The immune system is also composed of immune organs, immune cells and immune molecules substances and of importance to triggering an antimicrobial response to maintain a relatively stable internal environment. Immune cells are composed of macrophage, dendritic cells (DCs), NK cells, T cells, B cells, CTLs, Myeloid-derived suppressor cells (MDSCs), CAR T cells etc. The innate immune system can identify and fight foreign pathogens. For example, DCs, macrophages, and mast cells perform immune surveillance and release immune mediators when the host is attacked. However, the adaptive immune system requires DCs and NK cells to ingest foreign antigens and to remember previous attacks^59^. Upon identification of PAMPs and DAMPs by PRRS, inflammasomes are activated, which facilitate inflammatory factors maturation, including IL-1β and IL-18, and N-terminal fragment formation through proteolysis of GSDMs. It lyses cells and causes pyroptosis, and immune cells (such as NK cells, CD8^+^ T cells, B cells, CTLs, CAR T cells, Macrophage, etc.) are subsequently recruited to protect the host, accompanied by increased immune activity^60^. Nevertheless, pyroptosis can serve to suppress immunoreaction and the mechanisms of pyroptosis in immune regulation are still not clear^60^.

The obscure tie between immune cells and pyroptosis seem to be related to inflammasomes, GSDMs and inflammatory factors, such as HMGB1, IL-1β and IL-18. For example, following the NLRC4 inflammasome activation in DCs through recognition of flagellin, noncognate memory CD8^+^ T cells are activated to promote interferon-γ (IFN-γ) secretion, accompanied by release of IL-18^61^. Interestingly, recent research has shown that during Salmonella infection, the NLRP3 inflammasome is inhibited, which reduces CD4^+^ T cells activity, suggesting that inflammasomes may be a potential target for immunotherapy^62^. Recently, researchers have found that, in breast cancer, the GSDME expression level is increased and caspase-8 can cleave GSDME, triggering pyroptosis and increasing DCs, CD4^+^ and CD8^+^ T cells^63^. Moreover, researchers have revealed that the expression of GSDMB have a relationship with CD4^+^ T cells and neutrophils in clear cell renal cell carcinoma, suggesting that GSDMB shows potential in controlling immune infiltrates^64^. It has been reported that IL-18 released by pyroptotic cells recruits or differentiates some immune cells, including NK cells, T cells, and monocytes, to regulate the immune system^65^. Following the cleavage of pyroptotic cells, however, HMGB1 is released and inhibits immune activity by facilitating MDSCs accumulation that are implicated in the inhibition of CD4^+^ and CD8^+^ T cells activation^66^. In addition, IL-1β contributes to recruiting monocytes and producing macrophages, resulting in the decline of DCs and inhibiting the immune activity^67^. All the evidence embodies that pyroptosis has a dual role in immune cells and exerts effects on quantity and quality of immune cells. Taken together, pyroptosis can regulate immune cells through inflammasomes, GSDMs and inflammatory factors and these may have the potential to enhance immune response. Of note, some immune cells, including CTLs, NK cells, and CAR T cells, can promote or regulate pyroptosis though special proteins, such as neutrophil elastase and granzymes^48^-^50^.

### Pyroptosis affects anti-tumor immunity through the tumor microenvironment

When exerting anti-tumor immunity, pyroptosis is inextricably linked with the tumor microenvironment (TME). The cellular parts of the tumor immune microenvironment (TIME) mainly contain tumor cells, endothelial cells and immune cells, among which the most relevant to anti-tumor activity is immune cells^68^. CTLs and NK cells can inhibit the development of tumor cells, while infiltrating immune cells such as macrophages and neutrophils can support tumor growth and escape. Regulatory T cells and MDSCs can inhibit immune response on the surface of tumor cells^69^.

Currently, it is revealed that pyroptosis can neutralize tumor immunity though TME (Table 1). On the one hand, pyroptosis affects anti-tumor immunity by releasing various cytokines. When tumor cells undergo pyroptosis, it is often attended by the release of the inflammatory cytokines IL-1β and IL-18, which possess pro-tumor and tumor-suppressive effects^70^. IL-18 takes a significant role in tumor growth, angiogenesis, invasion, and metastasis. It has been reported that IL-18 induces the differentiation of T-helper 1 (Th1) and T-helper 17 (Th17) cells, resulting in anti-tumor effects^71^. IL-18 modulates both innate and adaptable immune responses by recruiting or differentiating NK cells, T cells, monocytes, and other immune cells, hampering the growth and metastasis of various types of tumors. IL-18 also increases interferon (IFN) release and the killing roles of CTLs, neutrophils, and NK cells^65^. On the other hand, granzymes can activate certain GSDMs family proteins to affect anti-tumor immunity. GZMA can activate GSDMB, while GZMB and caspase-3 can activate GSDME^49^, ^72^. CTLs can induce pyroptosis in human GSDME or GSDMB-sensitized tumor cells. Cytolysis causes target cell death, and pyroptosis may enhance the killing effect of cytotoxic T lymphocytes in cancer cells. In particular, pyroptosis induction overcomes immunosuppression and activates systemic anti-tumor immunity. Tumor cells will produce a great number of neoantigens that stimulate the responses of systemic immune and conspicuously inhibit the tumor progression, which together with tumor cell eradication, is an important pathway to achieve long-term tumor control^73^.

Tumor cells often express immune checkpoint molecules that suppress T cells' function in tumors. For example, the interaction of programmed death ligand 1 (PD-L1) and programmed death-1 (PD-1) on T cells inhibit target recognition. Despite the fact that neither blocking the PD-1-PD-L1 pathway nor the temporary pyroptosis induction could inhibit the growth of 4T-1 tumor alone, combination therapy exerts a great inhibitory effect on tumor growth. Furthermore, human GSDMB expresses PD-L1 in mouse colon cancer and melanoma cells without affecting tumor growth in immunocompetent mice. Nevertheless, it enhanced tumor growth inhibition by blocking immune checkpoints with anti-PD-1 antibodies^49^, ^74^. The effectiveness of current immunotherapies depends on the existence of anti-tumor immunity, and the suppressive TIME inhibits the ability of T cells to fight tumor cells. Abnormalities in tumor vasculature can create physical barriers to T cell trafficking, which is detrimental to tumor immunity. Therefore, the precondition of initiating anti-tumor immunity is the normalization of microenvironment^75^, ^76^.

#### Inflammasomes in anti-tumor immunity

Inflammasomes have become the focus of many researchers due to their significant effect on anti-tumor immunity. Just like inflammation, inflammasomes have a two-sided impact on anti-tumor immunity. Inflammasomes can both inhibit and facilitate tumor growth, differentiation, invasion, and metastasis, depending on the factors that are yet to be explored. The relatively mature role of inflammasomes in tumors and anti-tumor immunity are listed in Table 2.

Inflammasomes are of importance to the regulation of various tumors and there is a close association between the expression of inflammasomes and the prognosis of patients^89^, ^90^. The regulation of NLRP3 may be a potential tool to control tumor development due to the inhibitory effect of NLRP3 on the progression and development of cancer cells in liver cancer and gastric cancer. NLRP3 expression is down-regulated in liver cancer and closely related to prognosis, as evidenced by work, suggesting that NLRP3 exert effects on liver cancer^91^. Recent evidence has shown that pyroptosis triggered by the NLRP3 inflammasome inhibits gastric cancer progression, which may provide a new therapeutic approach for gastric cancer patients in the future^92^. Similar to the NLRP3 inflammasome in liver cancer, the AIM2 inflammasome also possesses similar anti-tumor effects. In liver cancer, the expression level of AIM2 is often decreased, and exogenous overexpression of AIM2 can suppress cancer cells progression and development by inducing pyroptosis and inhibiting the mTOR-S6K1 pathway^93^. Moreover, AIM2 expression level is also significantly decreased or severely absent in colorectal cancer, and expression of the AIM2 inflammasome is negatively correlated with colorectal cancer-specific death and disease recurrence in patients, suggesting that there is a negative relationship between the AIM2 inflammasome and colorectal cancer^94^. It has been shown that in breast cancer, the NLRC4 inflammation expression is upregulated, triggering inflammation and subsequently inducing tumor invasion^95^. Interestingly, Zhai et al. 96 has found that along with increased levels of IL‐1β, NLRP1 serves to facilitate tumor development by inhibiting caspase-2/9-mediated apoptosis reliant on caspase-3/7 in metastatic melanoma. Overall, these studies indicate that roles of inflammasomes in tumors are complex and related to the type of inflammasomes and tumor, and TME.

Inflammasomes can regulate immune cells and inflammatory factors, such as IL-1β and IL-18, to inhibit tumor growth, differentiation, and metastasis. Recent research has demonstrated the way inflammasomes protect against tumors by activating anti-tumor immunity through IL-1β and IL-18. Evidence embodies that the release of IL-1β by activated NLRP3 inflammasome can promote the expansion of CD8^+^ T cells and improve the activity of immune checkpoint blockers to regulate immune functions^97^. In agreement, Dania et al. 98 have found that IL-1β released by activated inflammasomes in DCs is beneficial to the hyperactivation of DCs and stimulated powerful CTLs responses to strengthen anti-tumor immunity, thereby eradicating tumors in whole organisms. Li et al.^99^ have shown that the NLRP3 inflammasome exerts effects on the anti-tumor process of the CD39 antibody. The release of IL-18 mediated by the NLRP3 inflammasome increases the expression level of effector T cells, enhancing the effect of anti-tumor immunity. Moreover, the NLRP3 inflammasome blockade suppresses head and neck squamous cell carcinomas (HNSCC) growth by downregulating IL-1βexpression and reducing MDSCs, PD-1 and tumor-associated Macrophages (TAMs) in head and neck squamous cell carcinoma^100^. Interestingly, the NLRP3 inflammasome expression is negatively correlated to the number of effective CD4^+^ and CD8^+^ T cells that exert effects on anti-tumor immunity. In metastatic breast cancer, the NLRP3 inflammasome inhibition delays tumor growth by reducing IL-1β in metastatic breast cancer^101^. Of note, the NLRP3 inflammasome inhibition during cancer treatment plays a synergistic in anti-PD-1 treatment. Apart from the NLRP3 inflammasome, other inflammasomes are of importance to suppressing tumor progression. Lin et al. 102 have found that NLRC4/neuronal apoptosis inhibitor protein 5 (NAIP5) activated by flagellin is thought to be involved in anti-tumor induced by CD8^+^ T cells. Chai et al. 103 have demonstrated that the combination of a DNA vaccine and AIM2 in H1 Nanoparticles is important for the expansion of CD8^+^ T cells to suppress tumor growth in renal carcinoma. In summary, inflammasomes inhibit tumor development by the regulation of immune cells or inflammatory factors and may be a target for therapy against tumors.

However, cancer cells can also evade anti-tumor immunity through immunosuppression induced by inflammasomes. A deeper understanding of immunosuppression induced by inflammasomes has important implications for new ideas in cancer therapy. In melanoma cell line B16-F10, the NLRP3 inflammasome promotes the MDSCs accumulation to counteract anti-tumor immunity^104^. In pancreatic ductal adenocarcinoma (PDA), the NLRP3 inflammasome is beneficial to the expansion of immune-suppressive macrophages that promote PDA growth^105^. Lu et al. 106 have found that the NLRP3 inflammasome exerts effects on the up-regulation of PD-L1 to facilitate lymphoma growth in diffuse large B cell lymphoma. What's more, the NLRP3 inflammasome blockade during cancer treatment plays an antagonistic role in anti-PD-L1 therapy due to an imbalance in T cell proportion^106^. The NLRP3 inflammasome appears to have different immunosuppressive effects depending on the cancer type, suggesting a connection with cancer type specificity. This suggests that targeting NLRP3 may be a potential way of augmenting anti-tumor immunity. The inflammasomes of pyroptosis have a dual effect on anti-tumor immunity, potentially offering a new outlook on tumor immunotherapy.

#### Gasdermin family in anti-tumor immunity

Increasing evidence proves that the GSDM family exert effects on anti-tumor immunity. Here, we will focus on the latest studies based on the GSDM proteins involved and the pyroptosis in anti-tumor immunity.

##### GSDME in anti-tumor immunity

Research on both in vitro and in vivo models has shown GSDME to have a tumor-repressive role. When GSDME was overexpressed, it resulted in a decrease in cancer cell proliferation, migration, colony formation and invasion, while its decrease had the opposite effect. In the latter, tumors in GSDME knockout mice were found to be growing faster than those found in their non-knockout counterparts^107^. This is enough to prove that GSDME has a tumor-suppressive effect.

**Figure 2:**
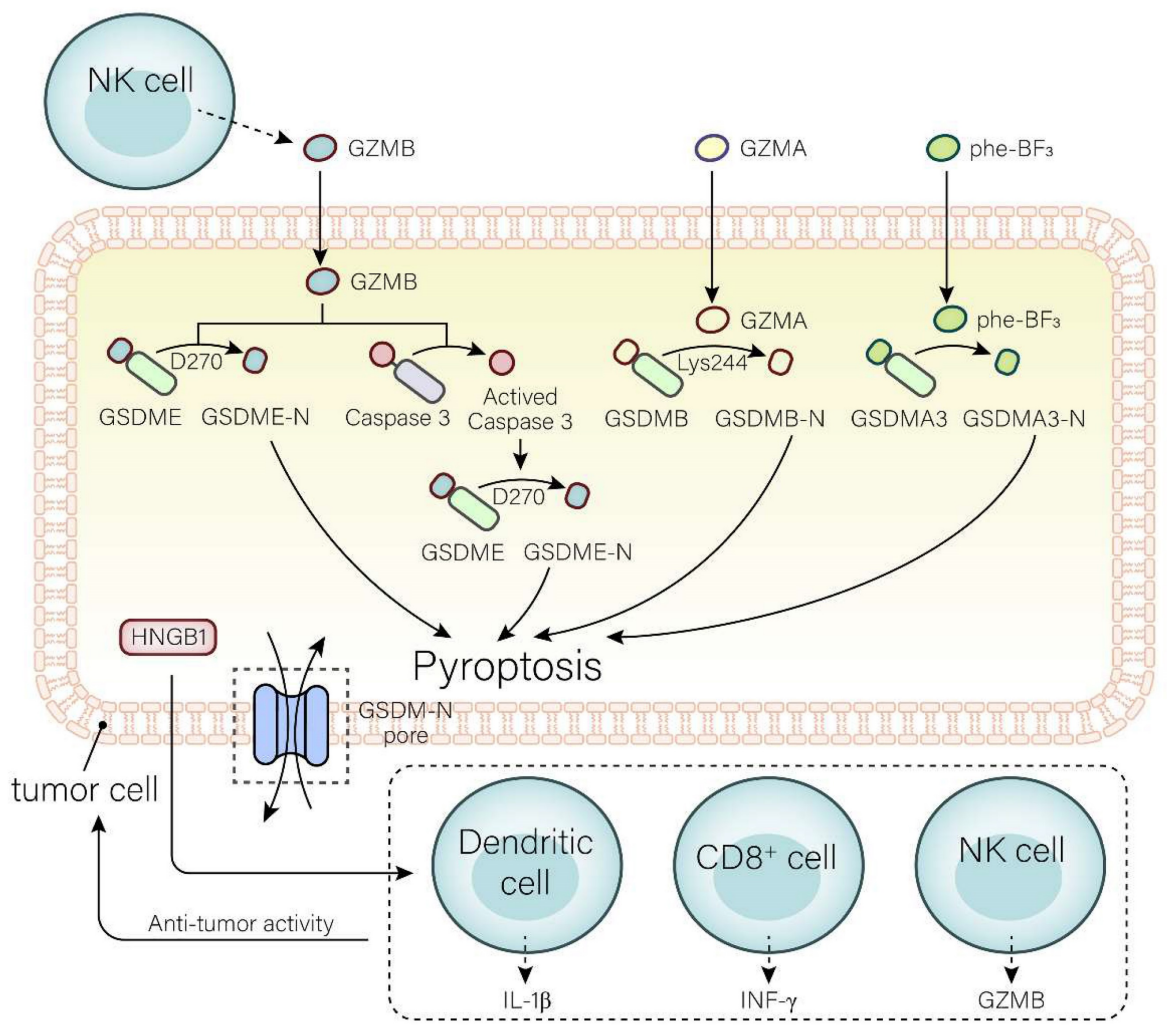
** Mechanisms of the gasdermin family involved in anti-tumor immunity.** In the GSDME pathway, GZMB directly cleaves GSDME to GSDME-N and GSDME-C at site D270 or activates caspase-3 to cleave GSDME into GSDME-N and GSDME-C. In the GSDMB pathway, GSDMB can be cleaved into GSDMB-N and GSDMB-C by GZMA at site Ly244. In the GSDMA3 pathway, extracellular Phe-BF3 enters the cell and cleaves GSDMA3 to GSDMA3-N and GSDMA3-C. GSDM-N produced by three pathways induces pyroptosis in tumor cells. The release of HGMB1 and GSDM-N produced in pyroptosis prompts the release of IL-1β, IFN-γ, and GZMB from DCs, CD8^+^ T cells, and NK cells to enhance the anti-tumor immune activity, and ultimately acts on tumor cells.

The tumor-suppressive function of GSDME can be reflected in three aspects (Fig. 2). Caspase-3 can be induced by intrinsic or external factors, like chemotherapy and drugs, resulting in GSDME-dependent pyroptosis. Anti-cancer drugs, such as pacisplatin, Lobaplatin, and triptolide, induce pyroptosis through the GSDME/caspase-3 pathway^108^-^111^. In addition, GSDME expression is associated with the proliferation and function of tumor-associated CD4^+^ T cells, CD8^+^ T cells, TAMs, NK cells, etc^50^. It has been demonstrated that GSDME expression is positively related to tumor-infiltrating CD8^+^ T cells, GZMB, and macrophages with an M1 phenotype^112^. The killing of target cells by CTLs is primarily triggered by the discharge of cytotoxic granules containing granzymes and perforin. NK cells release GZMB and activate GSDME by cleavage at D270. The expression of GSDME increases the phagocytosis of TAMs, enhances the function of tumor-infiltrating natural-killer, and then inhibits tumor growth^50^, ^72^. Furthermore, cleavage of GSDME and release of HMGB1 activate DCs, eventually leading to T cell proliferation, thereby exerting anti-tumor effects. Combined treatment with the v-raf murine sarcoma viral oncogene homolog B1 inhibitor, and MEK inhibitor may achieve the anti-tumor goal by promoting HMGB1 release and GSDME cleavage in DCs^113^.

##### The other members of gasdermin family in anti-tumor immunity

The GSDME is not alone in its efforts against anti-tumor immunity; other members of the GSDM family are just as important. If trifluorophenylalanine (PHF-BF3) is present, GSDMA3 induces tumor cell death and elicits potent antitumor immunity, mainly regulated by CD8^+^ T cells, thereby enhancing tumor clearance^74^, ^114^(Fig. 2). Wang et al.^74^ established a biorthogonal chemical system, a system based on demethylation of Phe-BF3, which was used to selectively control the release of GSDMA3 from nanoparticle conjugates in mouse 4T1 breast tumors. This model demonstrated that a mere 15% of the tumor cells undergoing pyroptosis was sufficient to eradicate the 4T1 breast tumor graft. Furthermore, the tumors remained in both immunodeficient and T cell-depleted mice, indicating that GSDMA3 triggers powerful anti-tumor immunity and possesses potent tumor suppressor properties.

GSDMB is highly expressed in tumor cells and is able to destroy them by triggering pyroptosis, lysing cells with GZMA, and releasing active pore-forming fragments. It is well known that cytotoxic lymphocyte-mediated immunity depends on granzymes. GZMA released by NK cells and CTLs can cause pyroptosis in GSDMB-positive cells. In addition, IFN-γ upregulates GSDMB expression and promotes pyroptosis^115^-^119^.

GSDMD is a major component in regulating pyroptosis. K3ZrF7:Yb/Er upconversion nanoparticles (ZrNPs), a pyroptosis inducer, induces tumor cell pyroptosis through the GSDMD/IL-1β pathway, leading to cell lysis. Pyroptosis induced by this pathway enhances the maturation of DCs, the frequency of effector memory T cells, and the inhibition of tumor growth, demonstrating superior antitumor immunity^120^. Su et al.^121^ constructed a carbonic anhydrase IX (CAIX)-anchored rhenium(I) photosensitizer (CA-Re) that activated GSDMD, triggering pyroptosis and an anti-tumor response. Simultaneously, it promoted DCs maturation and antigen presentation capacity, and completely activated T cell-dependent adaptive immune responses in vivo. Eventually, remote tumors were eliminated while destroying primary tumors.

### Pyroptosis in the clinical anti-tumor strategy

**Figure 3:**
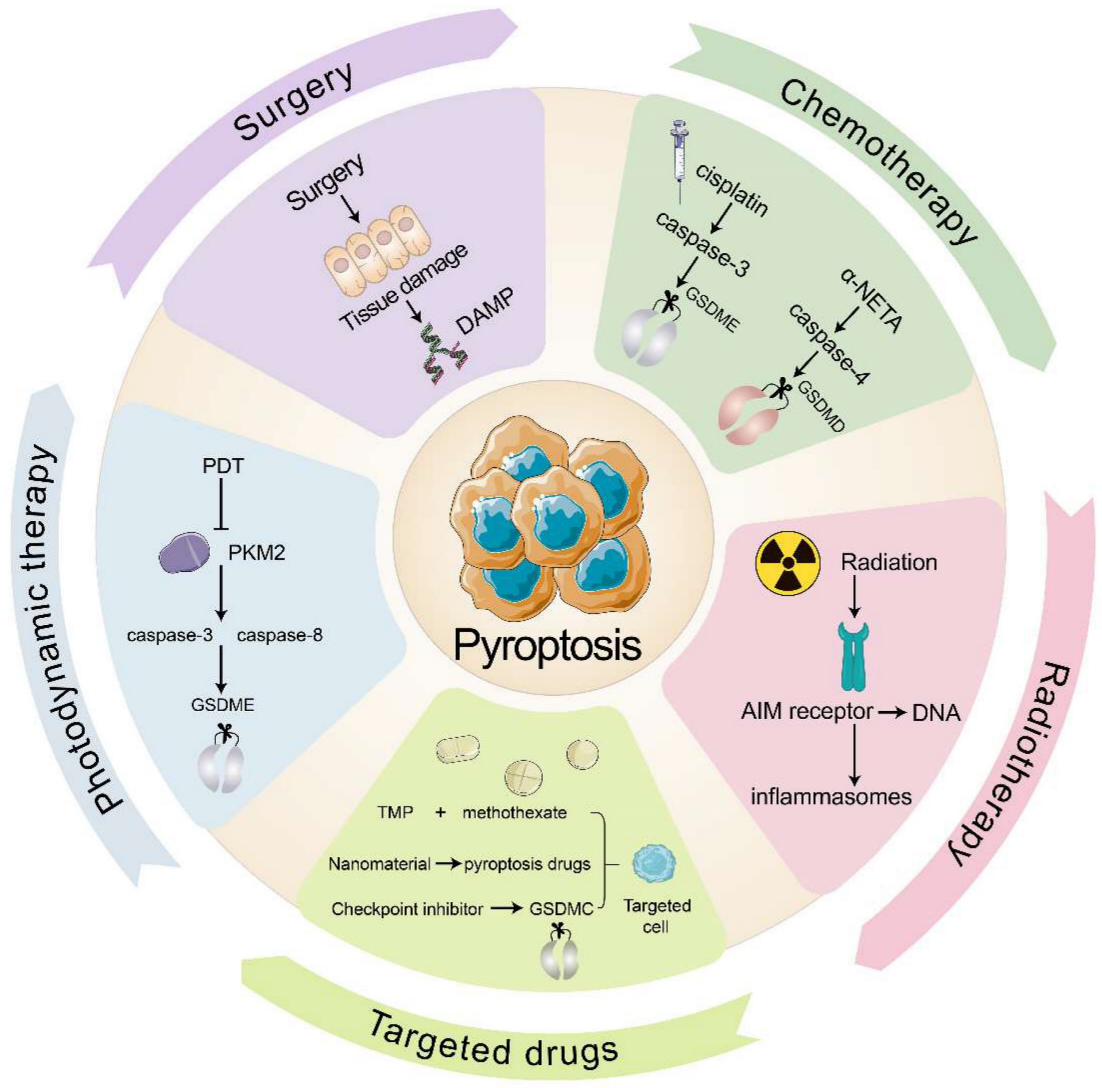
** Pyroptosis in the clinical anti-tumor strategy.** Surgery damages the tissue to release DAMP and then induces pyroptosis. Chemotherapeutic agents cisplatin and α-NETA induce pyroptosis through caspase-3/GSDME and caspase-4/GSDMD respectively. Radiotherapy allows the AIM receptor to recognize DNA by radiation, activate the inflammasome and induce pyroptosis. Targeted drugs induce pyroptosis in targeted cells. PDT inhibits PKM2 and induces pyroptosis through caspase-8, caspase-3/GSDME pathway.

In clinical practice, the primary anti-tumor therapies are surgery, chemotherapy, radiotherapy, immune checkpoint inhibitors, and targeted therapy. Several clinical cancer therapies also start from pyroptosis and TIME to promote tumor immunity (Fig. 3). Many chemotherapeutic drugs, including dihydroartemisinin (DHA), doxorubicin, and cisplatin, have been shown to induce pyroptosis and kill tumor cells (Table 3). In esophageal squamous cell carcinoma (ESCC), the pyruvate kinase M2 (PKM2)-caspase 8/3-GSDME pathway induces pyroptosis in ESCC cells^122^. Pyroptosis that doxorubicin induces in melanoma cells goes through the eucharyotic elongation factor 2 kinase (eEF-2K) /GSDME pathway. Inhibition of eEF-2K inhibits autophagy and enhances pyroptosis, thereby increasing the sensitivity of melanoma to doxorubicin^123^. Although most people believe that cisplatin kills tumors by apoptosis, recent evidence showed that cisplatin kills A549 cells by inducing pyroptosis through the caspase-3 / GSDME pathway, and that knockdown of GSDME reduced the sensitivity of A549 to cisplatin^108^. Taken together, these consequences reveal that pyroptosis may provide new insights into anti-tumor therapy.

Radiation therapy is an effective way to eliminate tumor cells with the help of high-energy radiation. Additionally, ionizing radiation can induce tumor immunity through pyroptosis, which is mediated by GSDME. Di et al.[124]has found that radiation causes pyroptosis that GSDME induces in nasopharyngeal carcinoma (NPC) cells, and the deubiquitination of GSDME enhanced the radiosensitivity of NPC. After pyroptosis occurs, cytokines begin to be released and CTLs appear. While radiation will kill immunogenic cells and promote anti-tumor immunity. Furthermore, ionizing radiation kill cells directly by destroying DNA. After AIM2 receptor recognizes DNA fragment, inflammasomes will be triggered, resulting in pyroptosis. Then inflammatory mediators liberating aggravate immune cells infiltrating in tumor and enhance anti-tumor immunity^125^.

In recent times, multiple studies have indicated that pyroptosis can be utilized in anti-tumor immunity, with the help of various drug targeting and delivery techniques. Nanomaterials have great benefits by diminishing their collection in healthy cells and adverse effects of targeted drugs. For example, the combination of cisplatin-containing tumor-targeting nanoliposomes and DNA methyltransferase inhibitors (DACs) inhibits DFNA5 gene methylation while activating caspase-3-mediated coagulation, thereby reducing tumor growth and transfer^83^, ^126^. In addition, tumor cell-derived microparticles (TMPs) also have a good targeting effect. TMPs inject methotrexate into cholangiocarcinoma cells (CCA), induce GSDME-mediated pyroptosis, activate patient-derived macrophages to produce pro-inflammatory cytokines, and recruit neutrophils to tumor sites for performing drug-directed tumor destruction^81^. Furthermore, the combination of various traditional and anti-inflammatory therapies may also provide new medical breakthroughs. For instance, photodynamic therapy (PDT) induces GSDME-mediated pyroptosis in ESCC. Mechanistically, PDT inhibits PKM2, which activates caspase-8 and caspase-3, eventually releasing GSDME-N and triggering pyroptosis in ESCC^127^.

To increase targeted immune responses to tumor antigens, immune checkpoint inhibitors are employed, with pyroptosis playing a major role. PD-1 and PD-L1 are the main targets of these inhibitors^128^. Clinical testing has demonstrated that a PD-L1 inhibitor coupled with chemotherapy or radiotherapy can bring about pyroptosis and put tumor cells to death. In comparison with PD-L1 inhibitors group, the survival of the combination therapy group has improved. This may be related to the effect of nuclear PD-L1 in promoting the transition from apoptosis to pyroptosis after GSDMC expression at the transcriptional level^129^, ^130^.

Lu et al.^131^ devised Natural Killer-92 (NK92) cells including a novel chimeric co-stimulatory transforming receptor (CCCR). This receptor could change PD-1/PD-L1 signal from negative to activated, enhancing the inhibiting immunity efficiency of PD-1. CCCR-NK92 cells were targeted to eliminate tumor cells through triggering pyroptosis in H1299 cells which is PD-L1-positive. If PD-1 and PD-L1 inhibitors play an initial role, the subsequent lesion induction will further improve the efficacy. At the same time, the inflammatory response changes the tumor microenvironment, thereby affecting the treatment of immune checkpoint inhibitors and making tumor immunity more sensitive. It can provide PD-1 and PD-L1 inhibitors along with other anti-tumor treatments. In conclusion, the prospect of pyroptosis in anti-tumor therapy is considerable. The continuous in-depth research and clinical application of pyroptosis will bring good news to the medical industry and patients.

## Conclusions and future perspectives

Pyroptosis is distinct from other cell death as it is associated with a powerful inflammatory response and cell rupture. We provide an in-depth explanation of the molecular process of pyroptosis, the function of the inflammasome and GSDMs, the relationship between pyroptosis and anti-tumor immunity, and the clinical applications of pyroptosis.

Pyroptosis is a major factor in the stimulation of anti-tumor immunity and the inhibition of tumors. However, the effects of pyroptosis on tumor cells are intricate. Pyroptosis activation in tumor and immune cells may promote the liberation of inflammatory chemicals, thus amplifying the efficacy of immunotherapy. At the same time, the activity of the inflammasomes and cytokines created by pyroptosis can result in chronic inflammation, which assists tumor cells in evading the immune system and advancing tumor progression. Current evidence indicates that pyroptosis is more likely to cause tumor promotion than inhibition, though different pathways can result in different cancers. Remarkably, reducing pyroptosis can actually promote tumor growth. It was discovered that a significant positive correlation exists between the downregulation of GSDMD and cancer cell proliferation. It is essential to delve deeper into the role of pyroptosis on the immune system, examining the association between other potential mechanisms and tumors to uncover more cancer therapies.

Recent evidence has revealed that particular virus-induced pyroptosis can lead to strong immune responses, for instance, NLRP3 was activated by SARS-CoV-2, prompting caspase-1 to induce pyroptosis in human monocytes. Moreover, encephalomyocarditis virus, an RNA virus that causes potassium outflow, activates the NLRP3 inflammasome in other cells to cause pyroptosis. Appropriate regulation of viral infection may be advantageous for tumor immunotherapy.

If pyroptosis induction is not managed correctly, it can harm the healthy tissue near the tumor. Nanomaterials may be a potential answer. By combining pyroptosis inducers and certain antibodies on the surface of nanoparticles, the delivery of nanoparticles in the TME can be improved, targeting cancer cells more accurately. This lowers the side effects of pyroptosis activation and allows for a more efficient distribution of inducers in the TME. The utilization of pyroptosis in a new manner is predicted to dramatically improve the effect of the treatment.

The mechanisms of action of GSDM family in pyroptosis have been gradually uncovered, yet the exact mechanisms of other GSDM families apart from GSDMD remain obscure. Notably, GSDMC has not been identified as a GSDMC-associated disease as its expression is limited. It has been reported that under hypoxic conditions, nuclear PD-L1 increases GSDMC expression, which is linked to poorer outcomes. This suggests that pyroptosis of chronic tumor cells in the central hypoxic zone, induced by GSDMC, may contribute to tumor progression. Despite this, the exact process remains unknown and requires further investigation.

Exploring the use of nanomaterials to induce pyroptosis is an area of development in the realm of anti-tumor immunity. Current strategies to induce pyroptosis come with a number of disadvantages, including severe systemic adverse effects, low bioavailability of drugs, limitation of immunosuppressive TME, and ineffective induction of immunogenic cell death. Fortunately, nanomaterials have been developed to address these issues, providing benefits like prolonged blood circulation, controlled drug release, and low toxic side effects. Additionally, a nanotherapeutic system that combines internal and external synergistic stimulation can induce pyroptosis in tumor cells, thereby activating a powerful systemic anti-tumor immune response that eliminates primary tumors and produces a memory effect to prevent metastasis of distant tumors^135^. Additionally, pyroptosis has pro-cancer and anti-cancer effects, but in the stage of cancer development, its anticancer effect is stronger than that of primary cancer^136^. It is crucial to grasp the balance between different tumor types and stages to achieve the best results in anti-tumor immunotherapy.

Several issues remain unresolved. Initially, the design of new nanomaterials is necessary, and further research is needed to comprehend the molecular mechanism of nanomaterials-induced pyroptosis and its potential consequences. Moreover, the changes in cellular functional changes caused by nanomaterials-mediated pyroptosis and signaling pathways have not been evaluated^137^. Additionally, the irregular expression and functioning of pyroptosis-related components is a major issue that needs to be addressed in different cancers or within cancers^138^. We anticipate that these issues will be resolved in the future as technology advances and molecular, genetic and epigenetic targeting/delivery systems become more precise and personalized.

## Figures and Tables

**Table 1:** The role of pyroptosis in cancer immunotherapy

Cancer type	Pyroptosis signaling	Immune factors	Increased Immune cells	References
Breast cancer	GSDMA3; NLRP3; GSDMD-mediated	Increase IL‐1β; IL‐18; HMGB1; IFNg; Gzm‐A, Gzm‐B; Gzm‐K; Fasl; IFN-γ. Decrease TAN, MDSC, and TAM populations.	Macrophage; CD4^+^T cells; CD8^+^ T cells; NK cells; CTLs; Long‐term memory T cell; leukocyte; DCs	63, 74, 77
Non‐small cell lung cancer cells	Caspase-1; caspase-3-mediated	Increase IL‐1β	RAW264.7 cells; peritoneal macrophages; THP‐1 cells; Tfh cells	78-80
Cholangiocarcinoma	Caspase-3-mediated	Increase IL‐1β	Macrophages	81
Melanoma	Caspase-1-mediated	Increase HMGB1	CD4^+^ T cells; CD8^+^T cells	82
Colon adenocarcino	Caspase-3-mediated	Increase IL‐1β and IL‐18	CD3^+^T cells; CD4^+^ T cells; CD8^+^T cells; CTLs; DCs	83
Cervical cancer	GSDMA3-mediated	Increase IL‐1β; IL‐18; HMGB1;	CD4^+^ T cells; CD8^+^T cells; NK cells;	74
Acute leukemia	Caspase-3; caspase-8; GSDME-mediated	Increase IL‐1β and IL‐18	THP-1 cells	84, 85
Gastric cancer	Caspase-3; GSDME-mediated	Increase TNF and IL‐17	CD4^+^T cells; CD8^+^ T cells; NK cells; TH1 cells	86, 87
Kidney Renal Clear Cell Carcinoma	Caspase-3; caspase-1; GSDME-mediated	Increase IL‐1β and IL‐18	CD4^+^T cells; CD8^+^ T cells; Tfh cells;Treg cells	88

**Table 2:** The role of inflammasomes in tumors and anti-tumor immunity.

Cancer types	The function of inflammasomes	References
Liver cancer	The NLRP3 inflammasome resists liver cancer development. The AIM2 inflammasome suppress the proliferation and metastasis of cancer cells by inducing pyroptosis and inhibiting the mTOR-S6K1 pathway.	91 93
Gastric cancer	The NLRP3 inflammasome resists the development of cancer cells.	92
Colorectal cancer	The expression of AIM2 inflammasome is negatively correlated with colorectal cancer-specific death and disease recurrence in patients.	94
Breast cancer	The NLRC4 inflammation triggers inflammation and subsequently induces tumor invasion. The NLRP3 inflammasome inhibition delayed tumor growth through the reduction in IL-1β.	95 101
Melanoma	NLRP1 serves to facilitate tumor development by inhibiting caspase-2/9-mediated apoptosis reliant on caspase-3/7 in metastatic melanoma. The release of IL-1β by activated NLRP3 inflammasome can promote anti-tumor immunity. The NLRP3 inflammasome promotes the accumulation of MDSCs to resist anti-tumor immunity.	96 97, 98, 104
B cell lymphoma	The release of IL-18 reliant on the NLRP3 inflammasome serve to stimulate anti-tumor immunity. The NLRP3 inflammasome is critical for the up-regulation of PD-L1 to promote lymphoma growth.	99 106
Renal carcinoma	DNA vaccine combined with AIM2 in H1 Nanoparticles is important to the expansion of CD8^+^ T cells to suppress tumor growth.	103
Pancreatic ductal adenocarcinoma	The NLRP3 inflammasome plays a vital role in the expansion of immune-suppressive macrophages to promote PDA growth.	105
Head and neck squamous cell carcinoma	The NLRP3 inflammasome blockade suppresses HNSCC growth by downregulating the expression of IL-1β and reducing MDSCs, PD-1 and TAMs.	100

**Table 3:** The role of chemotherapeutics in anti-tunor treatment.

Chemotherapeutics	Cancer types	Pyroptosis signaling	References
Paclitaxel	Lung cancer	Caspase-3/GSDME	108
α-NETA	Ovarian cancer	Caspase-4/GSDMD	132
Doxorubicin	Melanoma	eEF-2K/GSDME	123
Lobaplatin	cervical cancer	Caspase-3/GSDME	109
5-FU	Gastric cancer	Caspase-3/GSDME	86
Cisplatin	ESCC	Caspase-3/GSDME	133
Carboplatin	Retinoblastoma	Caspase-3/GSDME	134
DHA	ESCC	PKM2-caspase-8/3-GSDME	122

## Abbreviations

AIM2: absent in melanoma 2
ASC: apoptosis-associated speck-like protein containing a caspase recruit domain
CARD: caspase recruitment domain
CAR T cells: chimeric antigen receptor T cells
CA-Re: a carbonic anhydrase IX (CAIX)-anchored rhenium(I) photosensitizer
CCA: cholangiocarcinoma cells
CCCR: chimeric co-stimulatory transforming receptor
CTLs: cytotoxic T lymphocytes
DACs: DNA methyltransferase inhibitors
DAMPs: damage-associated molecular patterns
DCs: dendritic cells
DHA: dihydroartemisinic
DPP8: dipeptidyl peptidases 8
DPP9: dipeptidyl peptidases 9
eEF2K: eucharyotic elongation factor 2 kinase
ESCC: esophageal squamous cell carcinoma
GSDMB: Gasdermin B
GSDMC: Gasdermin C
GSDMD: Gasdermin D
GSDMD-C: a C-terminal domain of GSDMD
GSDMD-N: an N-terminal domain of GSDMD
GSDME: Gasdermin E
GSDMs: Gasdermins
GZMA: granzyme A
GZMB: granzyme B
HMGB1: high mobility group box 1
HNSCC: head and neck squamous cell carcinomas
IFN: interferon
IFN-γ: interferon-γ
IL-18: interleukin-18
IL-1β: interleukin-1β
IRAK: IL-1 receptor-associated kinase
LPS: lipopolysaccharides
MDSCs: Myeloid-derived suppressor cells
MyD88: myeloid differentiation primary response 88
NAIP5: neuronal apoptosis inhibitor protein 5
NF-κB: nuclear factor-kappa B
NK cells: natural killer cells
NK92: Natural Killer-92
NLR: NOD-like receptor
NLRC4: NOD-like receptor protein C4
NLRP1: NOD-like receptor family pyrin domain-containing 1
NLRP3: NOD-like receptor protein 3
NPC: nasopharyngeal carcinoma
PAMPs: pathogen-associated molecular patterns
PCD: programmed cell death
PD-1: programmed death-1
PDA: pancreatic ductal adenocarcinoma
PD-L1: programmed death ligand 1
PDT: photodynamic therapy
PHF-BF3: trifluorophenylalanine
PKC: protein kinase C
PKM2: pyruvate kinase M2
PKN1: Protein Kinase N1
PKN2: Protein Kinase N2
PRRs: pattern-recognition receptors
RhoA: Ras homolog gene family member A
RIPK1: receptor interacting protein 1
ROS: reactive oxygen species
TAMs: tumor-associated Macrophages
Th1: T-helper 1
Th17: T-helper 17
TIME: tumor immune microenvironment
TLR4: Toll-like receptor 4
TME: tumor microenvironment
TMPs: tumor cell-derived microparticles
TRF: time-restricted feeding
TTSS: type III secretion system
ZrNPs: K3ZrF7:Yb/Er upconversion nanoparticles
